# Risk and prognosis of secondary lung cancer after radiation therapy for thoracic malignancies

**DOI:** 10.1111/crj.13760

**Published:** 2024-05-09

**Authors:** Kang Chen, Chong Liu, Xueman Li, Tianyou Chen, Shan Liu, Fei Xiong, Zhou Zhang

**Affiliations:** ^1^ Wuhan Third Hospital & Tongren Hospital of Wuhan University Wuhan China

**Keywords:** radiation therapy, secondary lung cancer, thoracic cancer

## Abstract

**Objective:**

Radiation therapy (RT) may increase the risk of second cancer. This study aimed to determine the association between exposure to radiotherapy for the treatment of thoracic cancer (TC) and subsequent secondary lung cancer (SLC).

**Materials and Methods:**

The Surveillance, Epidemiology, and End Results (SEER) database (from 1975 to 2015) was queried for TC. Univariate Cox regression analyses and multiple primary standardized incidence ratios (SIRs) were used to assess the risk of SLC. Subgroup analyses of patients stratified by latency time since TC diagnosis, age at TC diagnosis, and calendar year of TC diagnosis stage were also performed. Overall survival and SLC‐related death were compared among the RT and no radiation therapy (NRT) groups by using Kaplan–Meier analysis and competitive risk analysis.

**Results:**

In a total of 329 129 observations, 147 847 of whom had been treated with RT. And 6799 patients developed SLC. Receiving radiotherapy was related to a higher risk of developing SLC for TC patients (adjusted HR, 1.25; 95% CI, 1.19–1.32; *P* < 0.001). The cumulative incidence of developing SLC in TC patients with RT (3.8%) was higher than the cumulative incidence (2.9%) in TC patients with NRT(P). The incidence risk of SLC in TC patients who received radiotherapy was significantly higher than the US general population (SIR, 1.19; 95% CI, 1.14–1.23; *P* < 0.050).

**Conclusions:**

Radiotherapy for TC was associated with higher risks of developing SLC compared with patients unexposed to radiotherapy.

## Graphical abstract

In this study, Cox regression analysis and multiple major standardized incidence ratios (SIRs) were used to assess the risk of SLC. Eventually, a certain number of relationship chains between radiation therapy for thoracic cancer (TC) and secondary lung cancer (SLC) were obtained from case data. The incidence risk of SLC in TC patients who received radiotherapy was significantly higher than in the general population (SIR, 1.19; 95% CI, 1.14–1.23; *P* < 0.050).

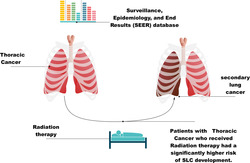

## 1 BACKGROUND

Breast cancer, lung cancer, and esophageal cancer are the three common primary thoracic cancers (TCs). The total incidence of TCs is more than 26.4% of diagnosed cancer.^1^ Radiation therapy (RT) is essential for the treatment of cancers for at least 40% of cancer patients undergoing its cure and improved prognosis.^2^ During RT, high dosages of ionizing radiation are delivered, which is a form of high‐energy electromagnetic radiation reaching deeper internal body structures and eventually causing apoptosis.^2^, ^3^

Radiotherapy is an important treatment to reduce the recurrence of these tumors and improve the prognosis.^4^, ^5^, ^6^ However, RT is also known to cause acute and late complications. One of the most serious late toxicity is a potentially increased risk of secondary tumors in the irradiated field.^7^, ^8^ As the patient's survival time increases, this elevated secondary cancer risk. Improvements in treatment for cancer over the past five decades have resulted in remarkable survival increases. Therefore, the long‐term adverse events of radiotherapy should be carefully considered.

Current studies have shown that in the abdominopelvic site, radiotherapy for in situ cancers leads to increased secondary cancers,^9^, ^10^, ^11^ and even radiotherapy affects the risk of cardiac death in patients with tumors,^12^ but there are no relevant studies in the chest. During RT for cancer of one of the thoracic organs, the lung is usually within the field of irradiation. Lung cancer as a second primary malignancy is increasingly common, but its risks are poorly understood.^13^, ^14^ Secondary cancer development is a multifactorial process, and the relationship between thoracic RT and secondary lung cancer (SLC) remains unclear. This study provides an in‐depth examination of the incidence and latency of SLC after RT for primary chest tumors based on actual cases.

## 2 MATERIALS AND METHODS

### 2.1 SEER database

The clinicopathological data of all female patients diagnosed with primary TCs were identified from nine registries of the Surveillance, Epidemiology, and End Results (SEER) database between January 1, 1973, and December 31, 2015, which is the population‐based registry for incident cancers in the United States and covers about 34% of the US population. SEER database collects and publishes information on cancer patient demographic profiles and cancer incidence, including race, primary tumor site, stage, second primary cancers, limited data on clinicopathological and treatment profiles, and survival data. This study has been approved by the Ethics Committee of Cancer Hospital, Wuhan Third Hospital & Tongren Hospital of Wuhan University.

### 2.2 Study population and outcomes

In this cohort study, solid TCs in three sites are routinely treated with radiotherapy (lung cancer, breast cancer, and esophagus cancer), which we defined as more than 20% of patients receiving radiation treatment as part of their first treatment course. Eligible female patients were between 20 and 85 years old and were diagnosed with TC (lung cancer [C19.9], breast cancer [C19.9], and esophagus cancer [C19.9]) according to SEER's International Classification of Disease (ICD‐O‐3). Localized, regional, and distant stage as defined by SEER was chosen for analysis. The included variables also included race, French Federation of Cancer Centers Sarcoma Group (FNCLCC) grade, multifocality, chemoradiotherapy, RT, extension of resection, follow‐up, and survival information. We excluded the samples with missing these variables from the analysis.

The primary outcome of this study was to evaluate the risk of developing a SLC with different treatment modalities. The female patients surviving less than 5 years were excluded, because the latency time between radiation exposure and solid‐cancer induction was at least 5 years.^1^ The follow‐up time for second cancers for each sample began 5 years after the date of diagnosis of the primary cancer and ended at the date of death, diagnosis of SLC, or after 30 years of follow‐up, whichever came first. It could help to eliminate surveillance bias that might result if patients who received radiotherapy were monitored more intensively than other patients in the first 5 years.^1^ The SEER program has eliminated the involvement of recurrent disease of TC according to the ICD‐O‐3 guidelines.

The secondary outcome was to estimate the 10‐year overall survival (OS), which was defined as the time from the start of SLC diagnosis to death due to any reason. We compare the survival of SLC patients receiving RT with SLC patients not receiving RT and SLC patients with patients diagnosed with only primary LC (OPLC). The OPLC is defined as the patient diagnosed with only primary LC during their lifetime.

### 2.3 Treatment interventions

SEER program collected information on the first course of treatment. According to the initial treatment modality of primary TC, patients could be classified into two groups. The RT group was composed of patients with TCs who received neoadjuvant external‐beam RT, and the no RT (NRT) group was composed of patients without RT. Patients receiving brachytherapy, radioisotopes, or combination RT were censored to decrease the bias caused by different types of RT.

### 2.4 Statistical analysis

The Cox proportional hazard regression analysis was performed with SLC as the event to evaluate the hazard ratios (HRs) and 95% confidence intervals (CIs) of developing SLC after TC. The multivariable Cox analysis was established by employing a backward selection procedure with variables with two‐sided *P* < 0.05 in univariable analyses, which were considered statistically significant and included in multivariable analyses. Fine‐gray competing risk regression analysis was employed to evaluate the cumulative incidence of SLC development. The SLC was considered as the event and a non‐SLC or all‐cause death were defended as competing events. *χ* ^2^ tests and the Fisher exact test were employed to compare categorical data.

The radiotherapy‐associated risk (RR) was calculated by Poisson regression analysis with the relative risk and 95% CIs of SLC development for TC patients receiving radiotherapy compared with those not receiving radiotherapy. These analyses were performed with R software (version 3.5.3). Besides, the standardized incidence ratio (SIR) and 95% CIs were also estimated by Poisson regression analysis. The definition of SIR was the ratio of observed developing SLC among TC survivors to the incidence of LC in the US general population. The SIRs were estimated with SEER*Stat 8.3.6. Both SIRs and RRs were adjusted with the age at TC diagnosis, race, and the calendar year of TC diagnosis. They were stratified by latency time since TC diagnosis, age at TC diagnosis, and calendar year of TC diagnosis.

The Kaplan–Meier method was used to determine 10‐year OS for SLC and OPLC, and survival differences were calculated by the log‐rank test. To decrease the potential bias for survival analysis, we performed propensity score matching (PSM) to match the cases and controls by using variables of age and year at SLC or OPLC diagnosis, race, stage of LC, tumor grade, and treatment for LC.

## 3 RESULTS

### 3.1 Demographic features

**FIGURE 1:**
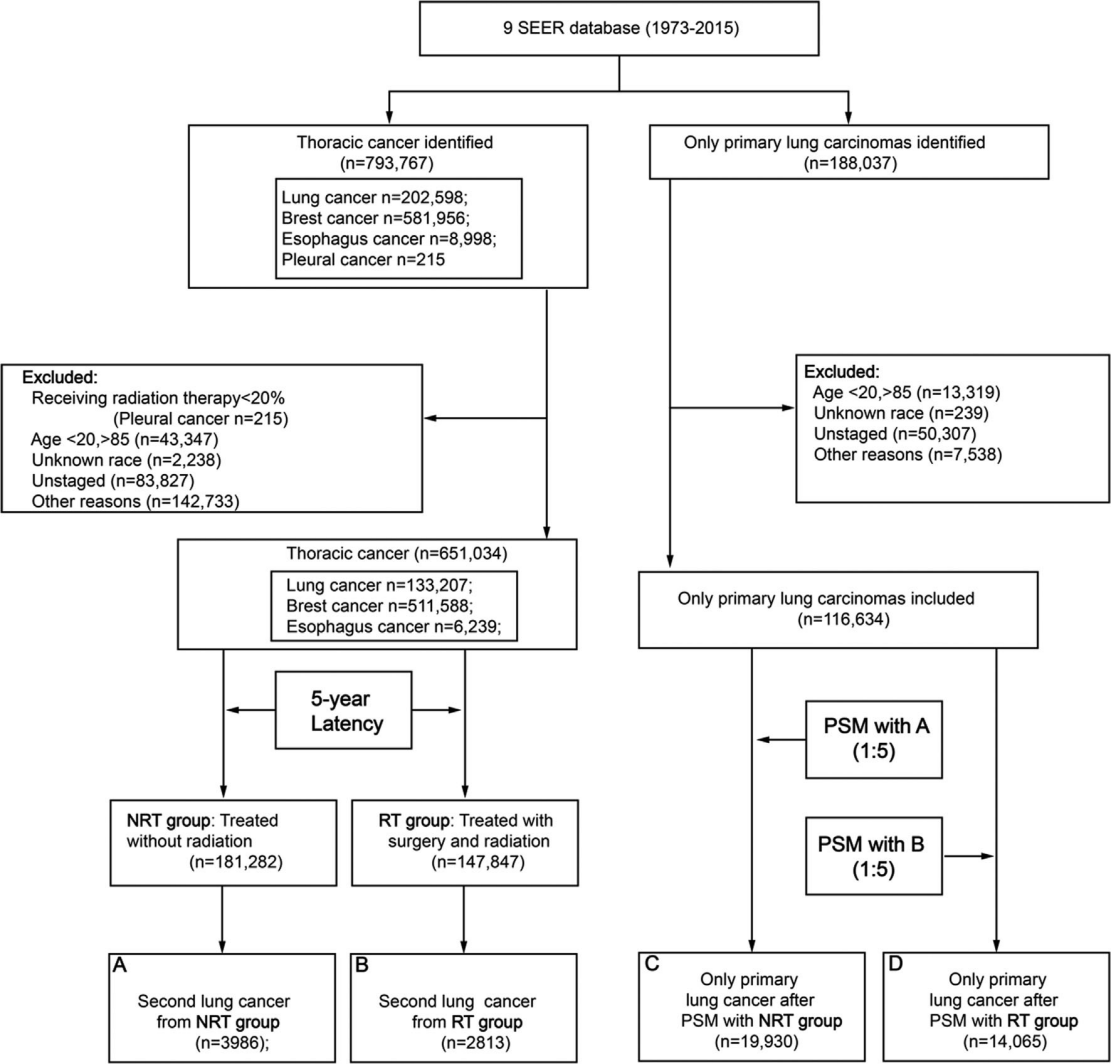
Flow diagram. RT, radiation therapy; NRT, no radiation therapy; SEER, Surveillance, Epidemiology, and End Results; PSM, propensity score matching.

A total of 793 767 patients with TC were identified in the cohort, and 329 129 patients remained after the exclusion of those with non‐matching information (Table 1 and Figure 1). 147 847 TC patients received RT, and 181 282 TC patients did not receive RT. The median follow‐up time was 136 months (interquartile range, 93–196 months) for the RT group and 151 months (interquartile range, 98–232 months) for the NRT group. The TC patients are significantly younger in the RT group (median 58 years old interquartile range, 49–67) than in the NRT group (median 60 years old interquartile range, 49–70). There were significant differences in age, race, grade, and stage between patients in the RT group and the NRT group. More patients in the RT group had received chemotherapy than in the NRT group; however, there were more patients with surgery in the NRT group than in the RT group. The proportion of TC patients who received RT as part of their initial treatment modality varied from 25.3% for lung cancer to 67.5% for esophagus cancer. The baseline characteristics of TC patients who developed an SLC were similar to the entire cohort.

**TABLE 1:** Comparisons of baseline characteristics of patients with thoracic cancer by treatment modality.

Characteristic	NRT (*n* = 181 282)	RT (*n* = 147 847)	*P*‐value
Tumor site, No. (%)			<0.001
Lung	12 607 (7.0)	4260 (2.9)	
Breast	168 419 (92.9)	143 180 (96.8)	
Esophagus	256 (0.1)	407 (0.3)	
Median age at TC diagnosis, (IQR), years	60 (49–70)	58 (49–67)	<0.001^a^
Age at TC diagnosis, No. (%), years			<0.001^b^
20–49	46 190 (25.5)	40 948 (27.7)	
50–69	87 293 (48.1)	77 841 (52.6)	
≥70	47 799 (26.4)	29 058 (19.7)	
Median year of TC diagnosis (IQR)	1993 (1985–2002)	2001 (1994–2006)	<0.001^a^
Year of TC diagnosis, No. (%)			<0.001^b^
1975–1984	41 934 (23.1)	9692 (6.6)	
1985–1994	56 110 (31.0)	27 786 (18.8)	
1995–2004	49 530 (27.3)	60 949 (41.2)	
≥2005	33 708 (18.6)	49 420 (33.4)	
Race, No. (%)			<0.001^b^
White	155 803 (85.9)	123 728 (83.7)	
Black	13 318 (7.4)	11 750 (7.9)	
Other	12 161 (6.7)	12 369 (8.4)	
Tumor grade, No. (%)			<0.001^b^
Grade I/II	66 861 (36.9)	79 315 (53.6)	
Grade III/IV	43 695 (24.1)	41 929 (28.4)	
Unknown	70 726 (39.0)	26 603 (18.0)	0.001^b^
Tumor stage, No. (%)			
Localized	121 875 (67.2)	97 482 (65.9)	
Regional	55 745 (30.8)	46 458 (31.4)	
Distant	3662 (2.0)	3907 (2.7)	
Surgery, No. (%)			0.833
No	4202 (2.3)	4350 (2.9)	
Yes	177 080 (97.7)	143 497 (97.1)	
Chemotherapy, No. (%)			<0.001^b^
No	139 118 (76.7)	86 811 (58.7)	
Yes	42 164 (23.3)	61 036 (41.3)	

*Note*: *P*‐value was calculated using the Mann–Whitney *U*‐test (^a^) for continuous variables and *χ* ^2^ test (^b^) for categorical variables.

Abbreviations: IQR, interquartile ratio; NRT, no radiation therapy; RT, radiation therapy; SLC, secondary lung cancer; TC, thoracic cancer.

### 3.2 Risk of SLC attributable to radiotherapy

**FIGURE 2:**
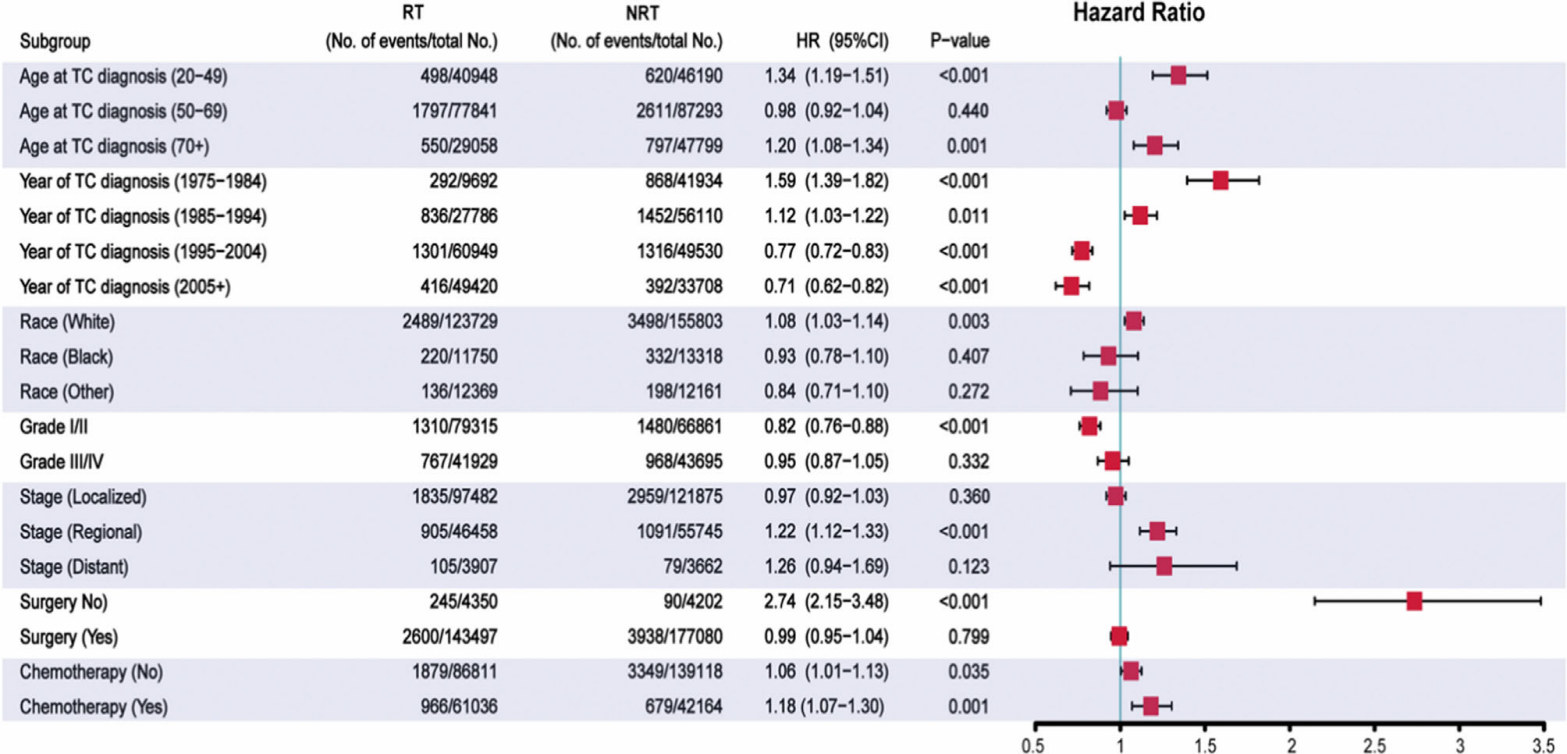
Subgroup analyses of Cox regression analysis for the risk of developing secondary lung cancer in primary TC patients.

All variables identified in Table 1 were selected for univariable Cox regression analysis to estimate the risk of developing SLC, and the variables, including age at TC diagnosis, year at TC diagnosis, race, tumor stage, tumor site, surgery, chemotherapy, and RT, could significantly influence the risk of developing SLC (with *P* < 0.05) (Table 2). In multivariable analysis, the variables, including age at TC diagnosis, year at TC diagnosis, race, tumor site, surgery, chemotherapy, and RT, could significantly influence the risk of developing SLC (with *P* < 0.05). And receiving radiotherapy was related to a higher risk of developing SLC for TC patients (adjusted HR, 1.25; 95% CI, 1.19–1.32; *P* < 0.001). To further estimate the risk of developing SLC, we performed the subgroup analysis for TC patients with Cox regression analysis. The results showed that the risk associated with RT increased significantly in most subgroups, but not in all (Figure 2). In analyses of each type of TC, receiving radiotherapy could significantly increase risks in breast cancer (adjusted HR, 1.26; 95% CI, 1.19–1.33; *P* < 0.001) and lung cancer patients (HR, 1.33; 95% CI, 1.18–1,49; *P* < 0.001) but not in esophagus cancer patients (Table 2), which suggested that the primary tumor site could influence the risk associated with RT.

**TABLE 2:** Univariable and multivariable Cox regression analysis of risk of developing second lung cancer in patients with thoracic cancer.

Characteristic (OS)	TC				LC				BC				EC	
	Univariable analysis		Multivariable analysis		Univariable analysis		Multivariable analysis		Univariable analysis		Multivariable analysis		Univariable analysis	
	HR (95%CI)	*P*‐value	HR (95%CI)	*P*‐value	HR (95% CI)	*P*‐value	HR (95%CI)	*P*‐value	HR (95%CI)	*P*‐value	HR (95%CI)	*P*‐value	HR (95%CI)	*P*‐value
Age at primary cancer diagnosis														
20–49	1		1		1		1		1		1		NA	NA
50–69	2.53 (2.37–2.70)	<0.001	2.26 (2.12–2.42)	<0.001	1.94 (1.62–2.33)	<0.001	1.99 (1.66–2.38)	<0.001	2.32 (2.16–2.49)	<0.001	2.26 (2.10–2.43)	<0.001	NA	NA
≥70	2.48 (2.29–2.69)	<0.001	2.16 (1.99–2.35)	<0.001	1.63 (1.33–1.99)	<0.001	1.73 (1.41–2.12)	<0.001	2.31 (2.11–2.52)	<0.001	2.21 (2.01–2.43)	<0.001	NA	NA
Year of primary cancer diagnosis														
1975–1984	1		1		NA		NA		1		1		1	
1985–1994	1.28 (1.19–1.37)	<0.001	1.02 (0.95–1.10)	0.542	1		1		1.11 (1.03–1.20)	0	1.06 (0.98–1.14)	0.149	0.65 (0.09–4.61)	0.665
1995–2004	1.58 (1.47–1.70)	<0.001	1.15 (1.06–1.25)	<0.001	1.44 (1.27–1.64)	<0.001	1.41 (1.24–1.61)	<0.001	1.21 (1.12–1.30)	<0.001	1.08 (0.98–1.19)	0.116	2.06 (0.46–9.15)	0.341
≥2005	1.87 (1.70–2.06)	<0.001	1.28 (1.16–1.42)	<0.001	1.78 (1.55–1.99)	<0.001	1.67 (1.42–1.97)	<0.001	1.21 (1.08–1.36)	<0.001	1.09 (0.95–1.24)	0.223	2.82 (0.57–14.02)	0.205
Race														
White	1		1		1		1		1		1		1	
Black	1.17 (1.07–1.28)	<0.001	1.20 (1.10–1.31)	<0.001	1.33 (1.13–1.57)	<0.001	1.31 (1.11–1.55)	0.001	1.07 (0.97–1.19)	0.2	1.17 (1.06–1.30)	0.003	1.07 (0.37–3.14)	0.9
Other	0.68 (0.61–0.75)	<0.001	0.70 (0.63–0.79)	<0.001	0.86 (0.69–1.09)	0.22	0.82 (0.65–1.03)	0.089	0.66 (0.58–0.75)	<0.001	0.68 (0.60–0.77)	<0.001	NA	NA
Tumor grade														
Grade I/II	1		NA	NA	1		NA	NA	1		1		1	
Grade III/IV	0.97 (0.92–1.03)	0.39	NA	NA	1.10 (0.99–1.24)	0.098	NA	NA	0.85 (0.80–0.92)	<0.001	0.96 (0.90–1.04)	0.322	1.48 (0.62–3.56)	0.381
Tumor stage														
Localized	1		1		1		NA	NA	1		1		1	
Regional	1.00 (0.95–1.06)	0.893	1.02 (0.96–1.08)	0.472	1.11 (0.99–1.24)	0.098	NA	NA	0.93 (0.87–0.98)	0	1.04 (0.98–1.12)	0.189	0.82 (0.34–2.02)	0.671
Distant	2.12 (1.83–2.46)	<0.001	1.07 (0.91–1.26)	0.434	1.13 (0.93–1.37)	0.209	NA	NA	1.09 (0.85–1.40)	0.5	1.22 (0.94–1.57)	0.129	0.96 (0.22–4.18)	0.952
Surgery														
No	1		1		1		1		1		NA	NA	1	
Yes	0.27 (0.25–0.31)	<0.001	0.85 (0.75–0.97)	0.016	0.77 (0.67–0.88)	<0.001	0.99 (0.84–1.16)	0.883	0.99 (0.74–1.32)	0.9	NA	NA	0.27 (0.25–0.31)	<0.001
Chemotherapy														
No	1		1		1		1		1		1		1	
Yes	0.82 (0.77–0.86)	<0.001	0.92 (0.86–0.98)	0.014	1.47 (1.31–1.65)	<0.001	1.28 (1.10–1.48)	0.001	0.77 (0.72–0.82)	<0.001	0.85 (0.79–0.92)	<0.001	0.90 (0.39–2.06)	0.806
Radiation therapy														
No	1		1		1		1		1		1		1	
Yes	1.05 (1.00–1.11)	0.036	1.25 (1.19–1.32)	<0.001	1.33 (1.18–1.50)	<0.001	1.16 (1.00–1.36)	<0.001	1.26 (1.20–1.33)	0	1.26 (1.19–1.33)	<0.001	1.65 (0.71–3.86)	0.248

*Note*: Cox regression analyses are used to calculate the hazard ratios (HRs) and 95% confidence intervals (CIs) for second lung cancer (SLC) in patients with thoracic cancer treated with RT versus patients not treated with RT. Covariables that are significant in univariable Cox regression analyses analysis (*P* < 0.05) are included in the multivariable analysis. NA indicates that the clinical feature could not be used in Cox regression analyses, because of sample counts.

Abbreviations: BC, breast cancer; CI, confidence interval; EC, esophagus cancer; HR, hazard ratio; LC, lung cancer; NRT, no radiation therapy; RT, radiation therapy; SLC, second lung cancer; TC, thoracic cancer.

### 3.3 Cumulative incidence of SLC

**FIGURE 3:**
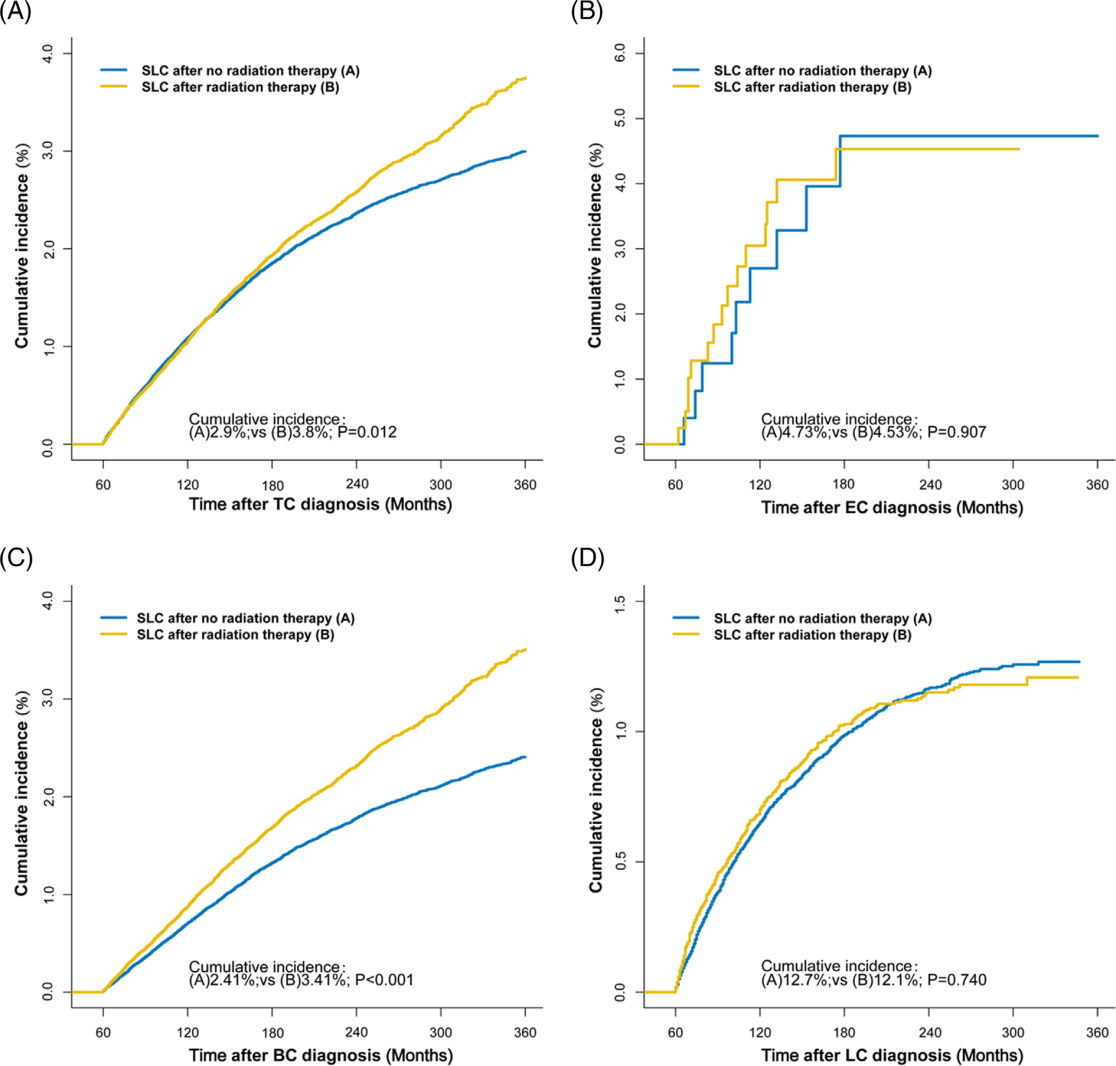
Comparisons of cumulative incidence of secondary lung cancer (SLC) between patients who received radiation therapy (RT) and patients who did not receive RT. *P*‐values were calculated with the Gray test.

The cumulative incidence of developing SLC was 3.16% after TC was diagnosed during 30 years of follow‐up, and the cumulative incidence curves separated with longer follow‐up. The cumulative incidence of developing SLC in TC patients with RT (3.8%) was higher than the cumulative incidence (2.9%) in TC patients with NRT (P) (Figure 3A). For different TC organs, the cumulative incidence of developing SLE was not consistent. The cumulative incidence of SLC in the breast was significantly higher in the radiotherapy group than in the non‐radiotherapy group (*P* < 0.001), but there was no difference observed in the lung and esophagus (Figure 3B–D).

### 3.4 RR and SIR of SLC

To evaluate the impact of RT on the risk of SLC, we further calculate RR based on the TC patients (Table S1). In multivariable analysis, the results showed that the adjusted RR of additional risk for SLC based on TC patients was 1.14 (95% CI, 1.09–1.28, *P* < 0.001), and radiotherapy was associated with a higher risk of developing SLC in organ‐specific analyses, including the increased RR noted in breast (adjusted RR, 1.29; 95% CI, 1.22–1.37; *P* < 0.001) and in lung (adjusted RR, 1.17; 95% CI, 1.04–1.32; *P* = 0.009). However, there was no difference observed in the esophagus.

Besides, we estimated the incidence risk associated with radiotherapy of developing SLC based on the general US population with SIR. The incidence risk of SLC in TC patients who received radiotherapy was significantly higher than the US general population (SIR, 1.19; 95% CI, 1.14–1.23; *P* < 0.050). On different TC tumor sites, SIRs for patients undergoing radiotherapy significantly promoted, including breast (SIR, 1.07; 95% CI, 1.03–1.11; *P* < 0.050), lung (SIR, 9.03; 95% CI, 8.04–10.10; *P* < 0.050), and esophagus (SIR, 2.97; 95% CI, 1.58–5.07; *P* < 0.050).

### 3.5 Dynamic risk and incidence evaluation for SLC

**FIGURE 4:**
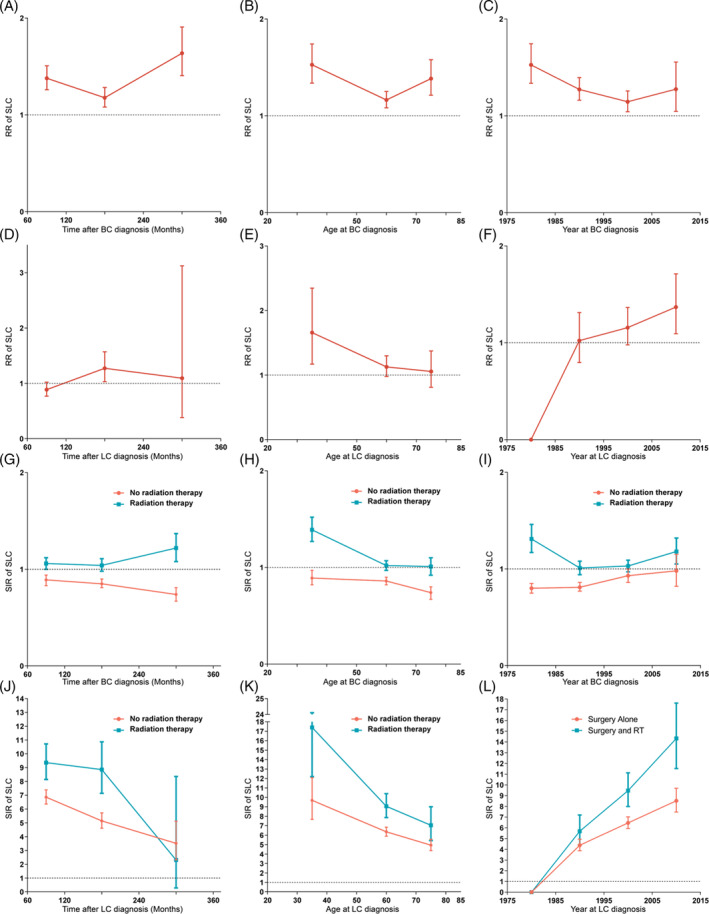
(A) Dynamic radiotherapy‐associated risk (RR) for secondary lung cancer (SLC) in latency‐RR plot; (B) dynamic RR for SLC in age‐RR plot; (C) dynamic RR for SLC in diagnosis time‐RR plot; (D) dynamic RR for secondary SLC in latency‐RR plot; (E) dynamic RR for SLC in age‐RR plot; (F) dynamic RR for SLC in diagnosis time‐RR plot; (G) dynamic standardized incidence ratio (SIR) for SLC in latency‐SIR plot; (H) dynamic SIR for SLC in age‐SIR plot; (I) dynamic SIR for SLC in diagnosis time‐SIR plot; (J) dynamic SIR for SLC in latency‐SIR plot; (K) dynamic SIR for SLC in age‐SIR plot; (L) dynamic SIR for SLC in diagnosis time‐SIR plot.

To estimate the dynamic incidence risk associated with radiotherapy of developing SLC, we performed three dynamic RR plots according to age at primary cancer diagnosis, latency period, and time of primary cancer diagnosis (Figure 4A). In the dynamic latency‐RR plot, the risk of SLC increased as the latency time went on in TC patients, and the risk significantly increased in the late latency (Figure 4B). The risk of SLC was also observed promoted in only latency among primary lung cancer patients and in late latency among breast cancer patients (Figure 4C). In the dynamic age‐RR plot, a conspicuously decreased risk of SLC was noted in TC patients, and the young TC patients were at high risk of developing SLC while undergoing radiotherapy (Figure 4D). The tendency of risk related to RT was similar in both breast and lung primary cancer patients. In the dynamic diagnosis time‐RR plot, the decreased risk of SLC was observed in TC patients from 1995 to 2016, and the risk associated with radiotherapy was still high from 1975–1994 (Figure 4E). The decreasing tendency of risk could be observed in primary breast cancer patients from 1975–2005, and the risk remained elevated up to the years from 2005 to 2015 (Figure 4F). The steady increased risk of SLC was noted in primary lung cancer patients.

Besides, we performed the dynamic SIR plots according to the age at primary cancer diagnosis, latency period, and time of primary cancer diagnosis. From the dynamic latency‐SIR plots, the incidence of SLC in TC patients receiving radiotherapy was higher than those in the US general population (Figure 4G). The similar tendency of SIR could be also observed in breast and lung cancer patients (Figure 4H). In the dynamic age‐SIR plots, the decrease in risk of SLC could be noted in different ages at TC cancer diagnosis groups undergoing RT (Figure 4I). Compared with the US general population in the matching age group, TC patients with RT were at a significantly higher risk of developing SLC, especially for the younger TC patients (Figure 4J). In the primary breast and lung cancer group, younger patients were in higher incidence of SLC than the older, which was similar with the RR (Figure 4K). In the dynamic diagnosis time‐SIR plot, the incidence of SLC was increasing significantly in the lung primary cancer group with RT, but the risk of reaching baseline rates could be noted in TC cancer patients with RT (Figure 4L). The details of RRs and SIRs are shown in Table S1.

### 3.6 Survival outcome of SLC

**FIGURE 5:**
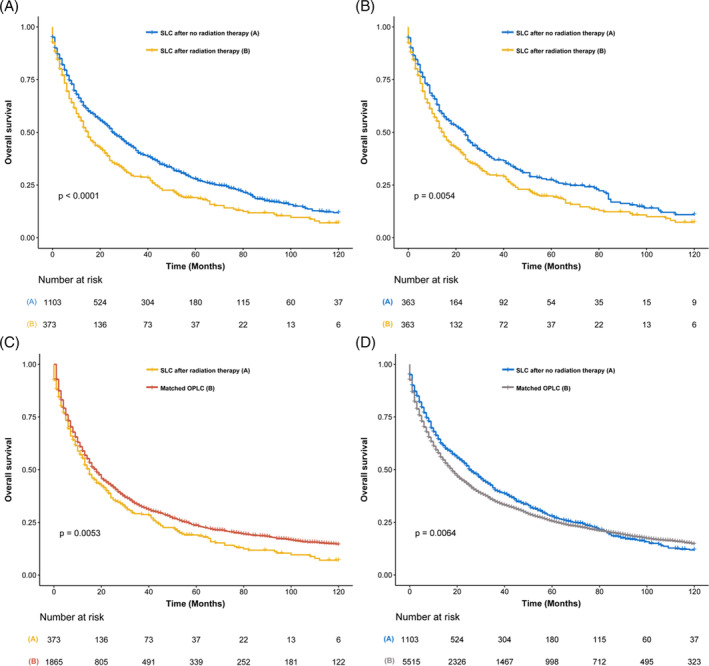
(A) Survival comparison between lung cancer (LC) patients who developed secondary lung cancer (SLC) after radiation therapy (RT) and LC patients who developed SLC after no RT (NRT) (before PSM); (B) survival comparison between LC patients who developed SLC after RT and LC patients who developed LC after NRT (after PSM); (C) survival comparison between LC patients who developed SLC after RT and patients with only primary lung cancer (OPLC); (D) survival comparison between LC patients who developed SLC after NRT and patients with OP LC.

For further analysis of the effect of radiotherapy on the survival of SLC, we compared survival between TC patients with undergoing radiotherapy and those without undergoing radiotherapy, and there were no significant differences between the 10‐year OS of patients developing SLC after radiotherapy and that of patients after no radiotherapy, both before PSM (Figure 5A) and after PSM (Figure 5B). However, the patients developing SLC after radiotherapy had worse 10‐year OS than patients after no radiotherapy in the primary lung cancer group (Figure 5B). To further estimate the survival outcomes of SLC, we then matched only primary lung cancer patients as the control group with SLC patients who had suffered primary lung cancer. Compared with matched population controls with OPLC, a significant difference of 10‐year OS was observed between patients who developed SLC after RT and matched OPLC (10‐year OS, 24.8% vs. 37.9%, *P* < 0.001; Figure 5C), and a significant survival difference of 10‐year OS was observed in patients without RT compared with matched OPLC (10‐year OS, 22.1% vs. 35.2%, *P* < 0.001; Figure 5D). Detailed information on OPLCs and survival analyses are shown in Table S2.

## 4 DISCUSSION

Our study is a large‐scale population‐based study that comprehensively assessed the risk of SLC in TC survivors and assessed the survival outcome of SLC. We found that the cumulative incidence of SLC in TC patients who received RT was higher than without RT. Second, the incidence of SLC in TC patients receiving RT is higher than that of the entire American population, and the risk of SLC after RT decreases with the extension of the diagnosis time and increases with the latency period. The younger patients receiving RT are more likely to develop SLC. Third, compared with SLC without RT, no difference in survival was observed in SLC after RT. In sum, thoracic RT is associated with a potentially increased risk for secondary tumors. Especially the lung, which is in close anatomical relation to several thoracic target organs, is likely to be in the radiation range and can consequently receive relatively high doses of radiation.

This is the first study using a large population‐based cohort to conduct a comprehensive investigation of the prognosis of lung cancer as the second primary malignancy revealing a distinct, time‐varying disease course. Our research has the following advantages: First, the findings were based on SEER, and a total of 329 129 thoracic tumors were included to avoid the selection bias imposed by single‐center studies or small‐sample studies. Second, the research period is nearly 43 years, which makes the conclusions more reliable. Third, competing‐risk proportional hazard regression was used to obtain unbiased estimates of the risk factors of SLC. Kaplan–Meier method and the COX proportional approach are the main analysis methods of traditional risk prediction models, which can only deal with one result and may produce biased results in the presence of competitive risks. However, in our study, the risk of competition is particularly important, because a considerable part of TC survivors usually die for other reasons before the development of SLC.

Previous studies on SLC have been mainly focused on the incidence rather than the risk factors.^15^, ^16^ The relationship between radiotherapy and SLC is also unclear. It has been reported that SLC can occur following radiotherapy for breast cancer, in the third decade after exposure than during the first two decades.^17^ The relationship between radiotherapy and SLC is related to whether the patient smokes or not and the dose of radiotherapy.^18^ In another article, survivors of initial primary lung cancer with radiotherapy likely had a low risk of metachronous SLC during the first 5 years of follow‐up.^19^ The risk of SLC after RT treatment for TC patients presented conflicting results; several reasons may affect the interpretation of the results, including the sample size of the cohort, the length of follow‐up, latency period selection, and the research method. The result of Hu, Z.G. was only a 5‐year follow‐up, and the occurrence of the second tumor was a long‐term complex process, which may be the reason for the different results. Henson, K. E and Taylor, C are consistent with our conclusions. These two studies mainly focused on breast cancer, and our data more comprehensively analyzed the relationship between thoracic tumor radiotherapy and SLC, including breast cancer, lung cancer, and esophageal cancer.

The risk of SLC after RT decreases with the extension of the diagnosis time, which may be associated with the RT treatment modality they had received. Over the past 30 years, technical improvement in radiotherapy and also reduced side effects.^20^ The risk increases with the latency period; this may be due to increased screening, improved medical imaging, and developed treatment strategies that may have greatly increased detection and prolonged patients' survival and thus lead to a higher risk of exposure to a subsequent cancer.^21^ The younger patients who receive RT have a longer survival period and are more likely to develop SLC. The results of esophageal cancer are not completely consistent with thoracic tumors. The reason may be that the incidence of esophageal cancer in the United States is low and the number of patients that can be collected for analysis is small. In addition, because of the limitation of the volume of data in the SEER database, it is difficult for us to compare with some indicators of normal people.

Our preliminary findings can help clinicians understand this disease better, and the results suggest that a long follow‐up time is needed for patients undergoing thoracic RT, especially for young patients. When the tumor has occurred for more than 10 years, patients are more likely to develop SLC.

Our study also has limitations. First, family history, smoking status, and body weight are the strongest risk factors for cancer, but relevant information is not available from the SEER database. Second, information about whether the patient is receiving radiotherapy is inaccurate. Only the initial treatment information of the tumor was recorded in the SEER database, and whether the delayed RT is performed in subsequent treatment is unknown. Therefore, this may lead to underestimation of the actual risk of SLC associated with RT. Third, our study cohorts were retrospectively collected over a long period, during which the treatment of tumors has been greatly improved. Regimens of radiotherapy for each patient were not available to obtain, which may influence more detailed conclusions.

## 5 CONCLUSIONS

In our study, we found that radiation for TC was associated with an increased risk of SLC based on a large population‐based cohort from the SEER database. More attention should be paid to the surveillance of SLC in RC patients, especially young patients.

## AUTHOR CONTRIBUTIONS

The idea of the study was contributed by Kang Chen and Chong Liu. The manuscript was mainly written by Kang Chen, Xueman Li, Tianyou Chen, Fei Xiong, and Shan Liu. The manuscript with constructive suggestions was revised by Kang Chen and Zhou Zhang.

## CONFLICT OF INTEREST STATEMENT

The authors have no conflict of interest to disclose.

## ETHICS STATEMENT

SEER database collects and publishes information on cancer patient demographic profiles and cancer incidence, including race, primary tumor site, stage, second primary cancers, limited data on clinicopathological and treatment profiles, and survival data. This study has been approved by the Ethics Committee of Cancer Hospital, Wuhan Third Hospital & Tongren Hospital of Wuhan University.

## Supporting information


**Table S1.** Risk of Developing Second Lung Cancer in Patients with Thoracic Cancer by Statistical Method **NOTE**. Poisson regression analyses were used to calculate the radiation‐attributed risk (RR) and 95% CIs of second lung cancer (SLC) for patients with different types of thoracic cancer with RT versus patients with NRT. Similarly, Poisson regression analyses were used to calculate the standardized incidence ratio (SIR) and 95% CIs of SLC for patients with RT and NRT versus the US general population. Both RR and SIR were adjusted for race, age at primary cancer diagnosis and calendar year of primary cancer diagnosis in our analysis. **Abbreviations:** RT, radiation therapy; NRT, no radiation therapy; CI, confidence interval; SIR, standardized incidence ratio; RR, radiation‐attributed risk; NS, no significance.
**Table S2.** Characteristics of LC Patients with Secondary Lung Cancer and Patients with Matched Only Primary Lung Cancer. **NOTE.** Primary lung cancer (LC) patients who developed secondary lung cancer (SLC) were matched with patients with only primary LC (OPLC) at a PSM ratio of 1:5 for LC patients versus OPLC patients. The matched variables for PSM included age at LC diagnosis, year of LC diagnosis, race, stage of LC, grade of LC and treatment type of LC. **Abbreviations:** RC, rectal cancer; RT, radiation therapy; NRT, no radiation therapy; LC, lung cancer; OPLC, only primary lung cancer; PSM, propensity score matching.

## Data Availability

The data that support the findings will be available in Surveillance, Epidemiology, and End Results at https://seer.cancer.gov/following an embargo from the date of publication to allow for commercialization of research findings.
