# Supplementary material for: Risk and prognosis of secondary lung cancer after radiation therapy for thoracic malignancies
Source: Clin Respir J. 2024 May 9;18(5):e13760. doi: 10.1111/crj.13760 (PMC11082536; doi:10.1111/crj.13760)
Supplement: Supplementary file 1 — Table S1. Risk of Developing Second Lung Cancer in Patients with Thoracic Cancer by Statistical Method NOTE. Poisson regression analyses were used to calculate the radiation‐attributed risk (RR) and 95% CIs of second lung cancer (SLC) for patients with different types of thoracic cancer with RT versus patients with NRT. Similarly, Poisson regression analyses were used to calculate the standardized incidence ratio (SIR) and 95% CIs of SLC for patients with RT and NRT versus the US general population. Both RR and SIR were adjusted for race, age at primary cancer diagnosis and calendar year of primary cancer diagnosis in our analysis. Abbreviations: RT, radiation therapy; NRT, no radiation therapy; CI, confidence interval; SIR, standardized incidence ratio; RR, radiation‐attributed risk; NS, no significance. Table S2. Characteristics of LC Patients with Secondary Lung Cancer and Patients with Matched Only Primary Lung Cancer. NOTE. Primary lung cancer (LC) patients who developed secondary lung cancer (SLC) were matched with patients with only primary LC (OPLC) at a PSM ratio of 1:5 for LC patients versus OPLC patients. The matched variables for PSM included age at LC diagnosis, year of LC diagnosis, race, stage of LC, grade of LC and treatment type of LC. Abbreviations: RC, rectal cancer; RT, radiation therapy; NRT, no radiation therapy; LC, lung cancer; OPLC, only primary lung cancer; PSM, propensity score matching. [file CRJ-18-e13760-s001.docx]

**Supplementary Table1.** Risk of Developing Second Lung Cancer in Patients with Thoracic Cancer by Statistical Method

| Primary cancer type | (RT vs NRT) | | (RT vs US general population) | | (NRT vs US general population) | |
| --- | --- | --- | --- | --- | --- | --- |
|  | Adjusted RR (95% CI) | P-value | Adjusted SIR (95% CI) | P-value | Adjusted SIR (95% CI) | P-value |
| Thoracic cancer | 1.14 (1.09-1.28) | 0.001 | 1.19 (1.14-1.23) | <0.05 | 1.09 (1.05-1.12) | <0.05 |
| Lung cancer | 1.17 (1.04-1.32) | 0.009 | 9.03 (8.04-10.10) | <0.05 | 6.06 (5.7-6.44) | <0.05 |
| Breast cancer | 1.30 (1.22-1.37) | <0.001 | 1.07 (1.03-1.11) | <0.05 | 0.84 (0.81-0.87) | <0.05 |
| Esophagus cancer | 1.09 (0.48-2.51) | NS | 2.97 (1.58-5.07) | <0.05 | 2.28 (1.04-4.33) | <0.05 |

**NOTE**. Poisson regression analyses were used to calculate the radiation-attributed risk (RR) and 95% CIs of second lung cancer (SLC) for patients with different types of thoracic cancer with RT versus patients with NRT. Similarly, Poisson regression analyses were used to calculate the standardized incidence ratio (SIR) and 95% CIs of SLC for patients with RT and NRT versus the US general population. Both RR and SIR were adjusted for race, age at primary cancer diagnosis and calendar year of primary cancer diagnosis in our analysis.

**Abbreviations:** RT, radiation therapy; NRT, no radiation therapy; CI, confidence interval; SIR, standardized incidence ratio; RR, radiation-attributed risk; NS, no significance.

**Supplementary Table2.** Characteristics of LC Patients with Secondary Lung Cancer and Patients with Matched Only Primary Lung Cancer.

| Characteristic | LC  (NRT)  (n=1103) | OPLC matched with LC  (NRT)  (n= 5515) | P value | LC  (RT)  (n=373) | OPLC matched with LC  (RT)  (n=1865) | P value |  |
| --- | --- | --- | --- | --- | --- | --- | --- |
|  |  |  |  |  |  |  |  |
| Age at LC diagnosis, No. (%), years |  |  | 0.911 |  |  | 0.997 |  |
| 20-49 | 10 (0.9) | 43 (0.8) |  | 7 (1.9) | 35 (1.9) |  |  |
| 50-69 | 360 (32.6) | 1805 (32.7) |  | 160 (42.9) | 804 (43.1) |  |  |
| ≥ 70 | 733 (66.5) | 3667 (66.5) |  | 206 (55.2) | 1026 (55.0) |  |  |
| Year of LC diagnosis, No. (%) |  |  | 0.984 |  |  | 0.886 |  |
| 1975-1984 | NA | NA |  | NA | NA |  |  |
| 1985-1994 | 9 (0.8) | 48 (0.9) |  | 3 (0.8) | 11 (0.6) |  |  |
| 1995-2004 | 264 (23.9) | 1322 (24.0) |  | 64 (17.2) | 325 (17.4) |  |  |
| ≥ 2005 | 830 (75.2) | 4145 (75.1) |  | 306 (82.0) | 1529 (82.0) |  |  |
| Race, No. (%) |  |  | 0.982 |  |  | 0.989 |  |
| White | 927 (84.0) | 4647 (84.3) |  | 316 (84.7) | 1580 (84.7) |  |  |
| Black | 120 (10.9) | 594 (10.8) |  | 37 (9.9) | 182 (9.8) |  |  |
| Other | 56 (5.1) | 274 (5.0) |  | 20 (5.4) | 103 (5.5) |  |  |
| Tumor grade, No. (%) |  |  | 0.999 |  |  | 0.997 |  |
| Grade I/II | 340 (30.8) | 1703 (30.9) |  | 85 (22.8) | 427 (22.9) |  |  |
| Grade III/IV | 275 (24.9) | 1374 (24.9) |  | 96 (25.7) | 482 (25.8) |  |  |
| Unknow | 488 (44.2) | 2438 (44.2) |  | 192 (51.5) | 956 (51.3) |  |  |
| Tumor stage, No. (%) |  |  | 1 |  |  | 0.989 |  |
| Localized | 434 (39.3) | 2170 (39.3) |  | 148 (39.7) | 732 (39.2) |  |  |
| Regional | 239 (21.7) | 1200 (21.8) |  | 77 (20.6) | 399 (21.4) |  |  |
| Distant | 286 (25.9) | 1431 (25.9) |  | 96 (25.7) | 480 (25.7) |  |  |
| Unknown | 144 (13.1) | 714 (12.9) |  | 52 (13.9) | 254 (13.6) |  |  |
| Surgery, No. (%) |  |  | 0.916 |  |  | 1 |  |
| No | 739 (67.0) | 3707 (67.2) |  | 284 (76.1) | 1419 (76.1) |  |  |
| Yes | 364 (33.0) | 1808 (32.8) |  | 89 (23.9) | 446 (23.9) |  |  |
| Chemotherapy, No. (%) |  |  | 0.941 |  |  | 0.906 |  |
| No | 802 (72.7) | 4001 (72.5) |  | 237 (63.5) | 1176 (63.1) |  |  |
| Yes | 301 (27.3) | 1514 (27.5) |  | 136 (36.5) | 689 (36.9) |  |  |
| Radiation, No. (%) |  |  | 0.866 |  |  | 1 |  |
| No | 679 (61.6) | 3377 (61.2) |  | 224 (60.1) | 1121 (60.1) |  |  |
| Yes | 424 (38.4) | 2138 (38.8) |  | 149 (39.9) | 744 (39.9) |  |  |

**NOTE.** Primary lung cancer (LC) patients who developed secondary lung cancer (SLC) were matched with patients with only primary LC (OPLC) at a PSM ratio of 1:5 for LC patients versus OPLC patients. The matched variables for PSM included age at LC diagnosis, year of LC diagnosis, race, stage of LC, grade of LC and treatment type of LC.

**Abbreviations:** RC, rectal cancer; RT, radiation therapy; NRT, no radiation therapy; LC, lung cancer; OPLC, only primary lung cancer; PSM, propensity score matching.
